# The Characteristics of Intestinal Bacterial Community in Three Omnivorous Fishes and Their Interaction with Microbiota from Habitats

**DOI:** 10.3390/microorganisms9102125

**Published:** 2021-10-09

**Authors:** Sheng Bi, Han Lai, Dingli Guo, Xuange Liu, Gongpei Wang, Xiaoli Chen, Shuang Liu, Huadong Yi, Yuqin Su, Guifeng Li

**Affiliations:** 1 Guangdong Province Key Laboratory for Aquatic Economic Animals, School of Life Sciences, Sun Yat-Sen University, Guangzhou 510006, China; bish@mail2.sysu.edu.cn (S.B.); laih5@mail2.sysu.edu.cn (H.L.); guodingli1987@126.com (D.G.); liuxg27@mail2.sysu.edu.cn (X.L.); wgongpei@163.com (G.W.); chenxli27@mail2.sysu.edu.cn (X.C.); liush276@mail2.sysu.edu.cn (S.L.); yihd3@mail2.sysu.edu.cn (H.Y.); suyq9@mail2.sysu.edu.cn (Y.S.); 2 Southern Marine Science and Engineering Guangdong Laboratory, Zhuhai 519000, China; 3 Guangdong Provincial Engineering Technology Research Center for Healthy Breeding of Important Economic Fish, Guangzhou 510006, China; 4 Zhongshan Ophthalmic Center, Sun Yat-Sen University, Guangzhou 510006, China

**Keywords:** microbial community, artificial fishery habitat, microbial ecology, omnivorous fish, 16S rRNA

## Abstract

Artificial fishery habitats have been extensively used for fishery resource protection and water habitat restoration, and they could attract a large number of omnivorous fishes to gather together. This study intended to reveal the relationship between bacterial communities in the habitats (water and sediment) and intestines of omnivorous fishes (*Oreochromis mossambicus*, *Toxabramis houdemeri* and *Hemiculter leucisculus*). Therefore, we investigated the bacterial communities of samples collected from intestines, water, and sediments in artificial fishery habitats via 16S rRNA metabarcoding high-throughput sequencing technology. The results showed that there were significant differences in the composition, core indicators, diversity and prediction functions in water, sediments, and intestinal microbial communities of the three omnivorous fish. The microbial diversities were significantly higher in habitats than in intestines. The analysis of similarity (ANOSIM) and nonmetric multidimensional scaling (NMDS) results indicated that the intestine microbial communities (*T. houdemeri* and *H. leucisculus*) were more similar to the water microbiota, but the intestine microbial communities (*O. mossambicus*) were more similar to the sediments. Source tracking analysis also confirmed that the contribution of habitat characteristics to omnivorous fish intestinal microorganisms was different; the sediment had a greater contribution than water to the intestinal microbiota of *O. mossambicus*, which was consistent with their benthic habit. Moreover, the functional prediction results showed that there were unique core indicators and functions between the bacterial community of habitats and intestines. Altogether, these results can enhance our understanding of the bacterial composition and functions about omnivorous fish intestines and their living with habitats, which have provided new information for the ecological benefits of artificial fishery habitats from the perspective of bacterial ecology and contributed to apply artificial fishery habitats in more rivers.

## 1. Introduction

Artificial fishery habitats are constructed to mimic characteristics of the natural habitats in aquatic environments and to extend the structural complexity of aquatic organisms in systems, where natural habitats are unavailable or absent [1]. Numerous studies have been performed to elucidate the role of artificial fishery habitats for fisheries management throughout the world, including to entice fishes and increase their abundance [2,3,4,5,6], to provide spawning substrates [7,8], and to offer shelter for juvenile fishes [9]. It had been reported that there were three omnivorous fishes (*Oreochromis mossambicus*, *Toxabramis houdemeri* and *Hemiculter leucisculus*) which had become the absolute dominant species in artificial fishery habitats of Youjiang [10,11] due to the extensive adaptability and strong fecundity with them [12,13,14,15]. As such, when the artificial habitats performed these functions, they were likely to inevitably affect the microbial community within the fishes’ intestines, water, and sediments. Several recent studies have also explored the effects of artificial fishery habitats on microbial diversity [16,17,18]. However, studies on the relationship between bacterial communities in artificial fishery habitats and intestines of omnivorous fishes are still scarce. Although it is known that changes in feed composition could affect the fishes’ intestinal and environmental microorganisms [19,20,21], microbial community differentiation among different host species and habitats in unfed aquaculture remains to be elucidated. This study was carried out in Youjiang River, a tributary of the Pearl River Basin, and it is still widely representative and can be popularized. The research results can contribute to other rivers by designing the artificial fishery habitat adapted to local conditions. The *Toxabramis houdemeri* and *Hemiculter leucisculus* are two common small fishes of Cyprinidae. They are widely distributed and live in the middle and upper layer of water, which are typical omnivorous fishes [12,13,15]. In addition, the tilapia (*Oreochromis mossambicus*) is also an omnivorous fish and belongs to Cichlidae, which has become one of the main aquatic products in China due to its rapid growth, strong fecundity, excessive yield, and high protein content [14,15,22].

There are considerable reports on fish intestinal microbiota [23,24,25], which play an essential role in digestion, metabolism, and immune system regulation [26,27,28,29]. Similar to mammals, the gut microbiota of fish can be recognized as an organ, in itself responsible for key physiological functions which aid in the health maintenance of its host [30]. For example, the fish intestines are colonized by a diverse community of symbiotic and pathogenic microbes that are involved in various physiological processes, including the promotion of immune system development and the production of enzymes related with digestion [31,32]. It is important to characterize the bacterial communities present in fish and understand what factors influence that composition [33]. Recent reports had showed that Proteobacteria were most abundant in some fishes, such as silver carp (*Hypophthalmichthys molitrix*), bighead carp (*Hypophthalmichthys nobilis*), shorthorn sculpin (*Myoxocephalus scorpius*), lumpfish (*Cyclopterus lumpus*) arctic flounder (*Liopsetta glacialis*), cod (*Gadus morhua*), and herring (*Clupea pallasii*) [34,35]. The gastrointestinal bacterial community of convict surgeonfish was dependent on the location within the gut and the age of the individual [36]. Both common carp (*Cyprinus carpio*) which were exposed to waterborne copper and zebrafish which were exposed to polystyrene microparticles displayed disturbances of the intestinal microbiota related to immunity, which increased their susceptibility to pathogens and inflammation (microbiota dysbiosis) [37,38,39]. It is becoming increasingly clear that the microbiome affects its host in more than one way [40]. Now, the progress has been made in biotic and gnotobiotic zebrafish models, defining a core microbiome and describing its role in development [41].

Since aquatic animals are continuously exposed to water, the structure and composition of their mucosal microbiota are strongly affected by the habitats [42]. Previous studies had demonstrated that the intestinal microbial composition of fishes, shrimps, and crabs were affected by the surrounding environment (e.g., water or sediments) [43,44,45] or feeding strategy [46,47]. However, most of these studies were carried out by changing the feed composition and geographical location, or through environmental stress. Indeed, few researchers have constructed complex systems based on artificial fishery habitats to study the impact on the omnivorous fish intestines. Therefore, host-associated microbiota that is influenced by habitats remains relatively unclear. We hypothesized that the bacterial communities of water and sediments would have a substantially higher microbiota diversity compared to the omnivorous fish intestines due to the complex systems of artificial fishery habitats.

In this study, we used 16S rRNA metabarcoding high-throughput sequencing [40,48,49] to investigate microbiota in the fish intestines, water, and sediments from artificial fishery habitats. This study aimed to characterize the relationship of bacterial community between the fish intestines and habitats. These results could provide novel evidence of the effects about artificial fishery habitats and enhance our understanding of the bacterial composition, diversity and predictive functions about omnivorous fish intestines.

## 2. Materials and Methods

### 2.1. Study Sites and Structure of Artificial Fishery Habitats

**Figure 1:**
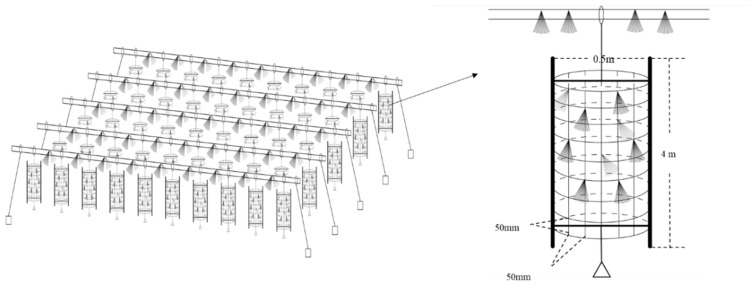
Schematic diagram of the artificial habitats [10].

The artificial fishery habitats were located between the cascade water control projects in the Youjiang river section of the Pearl River Basin, China (23.46° N, 106.41° E). In order to reduce the eutrophication of the water body, an unfed aquaculture program is being implemented in this river. More than 1000 structural units were constructed and laid on the experimental sites in an orderly way, covering an area of approximately 2000 square meters, which constituted the artificial fishery habitats (Figure 1). The artificial habitats had been applied to the experimental sites in December 2015 [10,11].

### 2.2. Sample Collection

Surrounding water, sediments, and fish intestines samples were collected from artificial fishery habitats. One sampling site was set every 200 square meters and 10 sampling sites covered the whole artificial fishery habitat areas. A total of 600 fish were captured, including *T. houdemeri* (200), *H. leucisculus* (200), and *O. mossambicus* (200). The large sample size excluded individual differences in experimental results. The fish surface was disinfected with 70% ethanol, while the intestines were aseptically removed from their abdominal cavity and placed into a 15 mL sterile centrifuge tube, every 20 fish as one sample. We sampled the entire intestine to minimize bias caused by the spatial structure of the gut microbiota [50]. Water samples (2000 mL) from the surface, middle, and bottom of the each site were mixed together in one sample and filtered through a 0.22 μm porous polycarbonate membrane (Millipore, Burlington, MA, USA) for DNA extraction [51]. Sediments samples were randomly collected three times at each site, and then mixed into a 15 mL sterilized centrifuge tube as one sample. All samples including fish intestines, filter membrane of water and sediments were immediately placed in liquid nitrogen and then stored at 80 °C until DNA extraction.

### 2.3. DNA Extraction, PCR Amplification, and 16S rRNA Sequencing

DNA was extracted from all samples (intestine, water and sediments) using a Bacterial DNA kit (MN NucleoSpin^®^ 96 Soil, Darmstadt, Germany) following the manufacturer’s instructions. The concentration and purity of genomic DNA were detected using a Nanodrop 2000c Spectrophotometer (Thermo Scientific, Waltham, DE, USA). In order to perform 16S rRNA gene amplification analysis, we amplified the V3-V4 hypervariable regions of the 16S rRNA gene using 338F (ACTCCTACGGGAGGCAGCA) and 806R (GGACTACHVGGGTWTCTAAT) primers [52,53]. Amplification followed a set procedure: denaturation at 94 °C (5 min), then 35 cycles at 94 °C (30 s), 53 °C (30 s), and 72 °C (30 s), with the final elongation at 72 °C (10 min). Amplicons were extracted from 2% agarose gels and purified using an AxyPrep DNA Gel Extraction Kit (Axygen Biosciences, Union City, CA, USA) according to the manufacturer’s instructions and quantified using an ABI StepOnePlus Real-Time PCR System (Life Technologies, Foster City, CA, USA). The purified PCR products of each group were pooled at equimolar concentrations. The libraries were sequenced with paired-end by an Illumina HiSeq2500 system (Illumina, San Diego, CA, USA) according to the standard protocols of Biomarker Technologies Co. Ltd. (Beijing, China). The raw data was uploaded to the Dryad Sequence Read Archive, and the download link is DOI: 10.5061/dryad.tqjq2bvxm, 20 November 2020.

### 2.4. Sequencing Data Processing and Statistical Analysis

To obtain high-quality reads, raw data were filtered using fastp, with the removal of reads containing more than 10% of unknown nucleotides or less than 60% of quality bases (Q-value > 20) [54]. Paired-end clean reads were merged using FLASH (Version 1.2.11) [55] with a minimum overlap of 10 bp and a mismatch error rate of 2%. The quality of the spliced sequences was filtered by Trimmmatic (version 0.3.3) [56], and chimeras were simultaneously removed by UCHIME (version 8.1) [57] to obtain high-quality 16S rRNA metabarcoding sequences. Effective tags were clustered into operational taxonomic units (OTUs) of more than 97% similarity threshold using the UPARSE (version 10.0) pipeline [58]. The representative sequence in each cluster from the tag sequence with the highest abundance was selected, and then the taxonomic information of groups were annotated for each representative sequence by the naive Bayesian model with the Ribosomal Database Project (RDP Release 11.5) classifier [59] which were based on the SILVA (version 132) database (http://www.arb-silva.de, 1 July 2021) [60].The alpha-diversity indices, including Shannon, Simpson, Chao-1 and ACE, were calculated from the OTUs of each library to estimate and compare the bacterial community diversity in each treatment [61]. Kruskal-Wallis tests were used to compare the bacterial diversity and OTU richness of water, sediment, and fish intestines; *p* < 0.05 indicated the significant difference. Deviations in bacterial community of intestines, water and sediments were visualized through NMDS analysis based on Bray-Curtis distances. The beta-diversity analysis of nonmetric multidimensional scaling (NMDS) based on the Bray-Curtis dissimilarity index of samples and the analysis of similarity (ANOSIM) were performed using the R program (“vegan” package) [62]. The contributions of different sources to the community composition of the fish intestines (source: WR, ST) were predicted with Fast Expectation-Maximization Microbial Source Tracking (FEAST) [63] Microbial community bar plots and Venn diagrams were produced using the free online platform BMKCloud (https://international.biocloud.net/zh/dashboard, 1 July 2021). In addition, the linear discriminant analysis effect size (LEfSe) can analyze effectively bacterial community data at the taxonomic phylum to genus levels. [64]. LEfSe analysis was using the online tool (http://huttenhower.sph.harvard.edu/galaxy/root?tool_id=lefse_upload, 1 July 2021). Functional changes in the bacterial communities between habitats and omnivorous fishes were predicted by using Phylogenetic Investigation of Communities by Reconstruction of Unobserved States (PICRUSt) [65]. We reconstructed the metagenome functional genes by rarefied 16S rRNA copy numbers, which were further classified via Kyoto Encyclopedia of Genes and Genomes (KEGG) categories at levels 1, 2 and 3 [66]. Furthermore, the genes which were the most differentially abundant functional between different samples were identified by using Welch’s *t*-test.

## 3. Results

### 3.1. Overview of the OTUs and Diversity Analysis

The 16S rRNA gene amplification products of all samples were detected through HiSeq sequencing and we obtained a total of 5,213,461 high-quality sequences after quality control and chimaera filtration. These sequences were clustered into 853, 1034, 1055, 1011, and 908 OTUs from the HI (*T. houdemeri* intestines), LI (*H. leucisculus* intestines), MI (*O. mossambicus* intestines), WR (water) and ST (sediment) groups, respectively (Table 1).

The alpha-diversity indices (Shannon, Gini-Simpson, Chao-1, and ACE) were calculated at the level of OTUs and results were showed in Table 1. The coverage was always maintained at 0.99 (Table 1). Shannon index and Gini–Simpson index are used to measure the bacterial diversity. The larger Shannon index and Simpson index indicated the higher bacterial diversity of the samples. Chao1 and ACE indexes are used to measure bacterial richness. The larger Chao1 and ACE indexes indicated the higher the bacterial richness of the samples. Results indicated that the WR samples showed the highest microbial diversity with the Shannon and Gini-Simpson index (Table 1). Furthermore, the microbial richness in the habitats samples (ST, WR) was significantly higher than in the intestines samples (LI, HI, MI) with the Chao1 and ACE indexes, the intestine microbial diversity of MI was higher than both LI and HI (Table 1).

**Figure 2:**
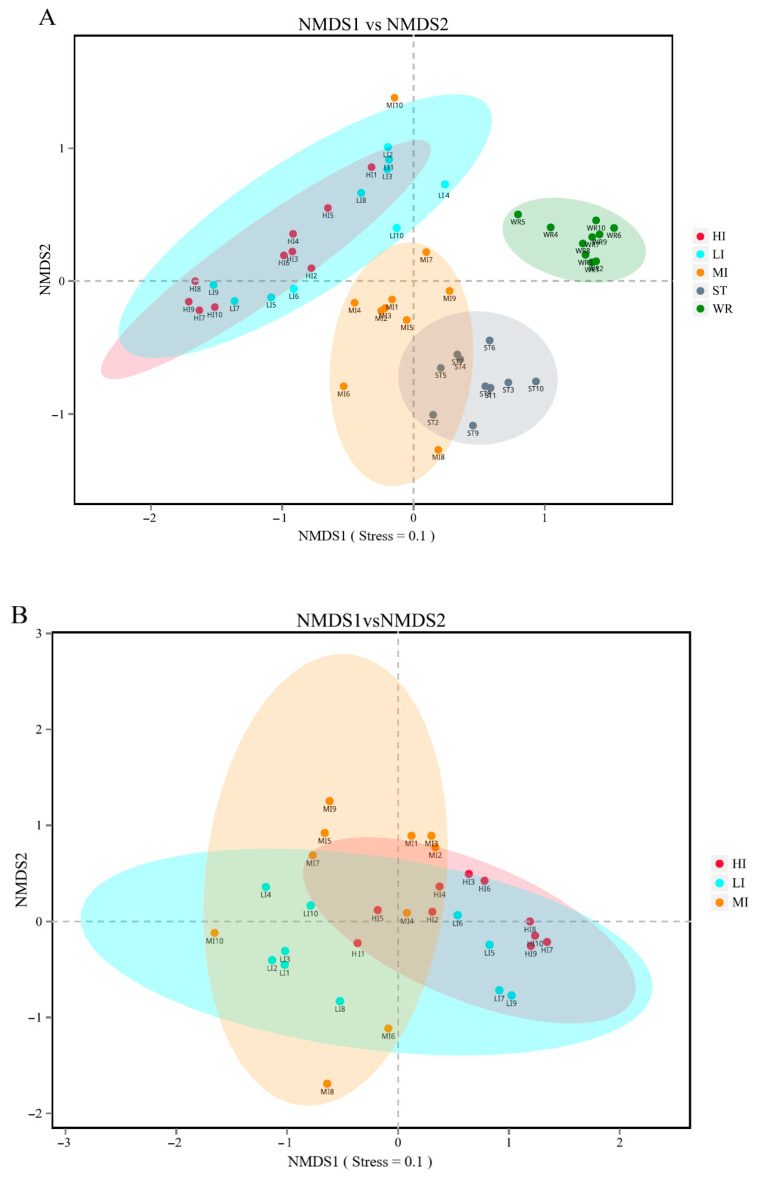

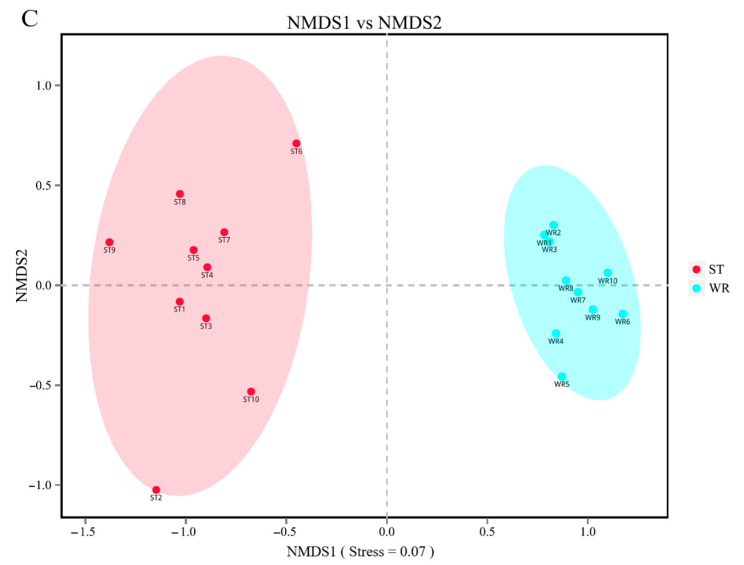
The results of the two-dimensional NMDS analysis at the level of OTUs. (**A**) The diversity analysis of habitats and fish intestines samples and (**B**) the diversity analysis of interindividual including HI, LI. and MI. (**C**) the diversity analysis of habitats including ST and WR. HI, *T. houdemeri* intestines; LI, *H. leucisculus* intestines; MI, *O. mossambicus* intestines; ST, sediment; and WR, water.

The bacterial beta-diversity was analyzed by NMDS based on the Bray-Curtis dissimilarity index of samples. The NMDS results showed that the water and sediments groups were separated from the fish intestine groups and were clustered independently, whereas the groups of LI and HI were generally clustered together and the MI group does not overlap with them, but with ST (Figure 2A). These results indicating that dissimilarity among the fish intestine groups was low, but dissimilarity between the habitat groups was high. Furthermore, the ANOSIM results (Table 2) showed that the WR/ST (0.75 < R < 1, *p* < 0.01) groups were well separated, whereas LI/MI (R = 0.4218, *p* = 0.008) and HI/MI (R = 0.4045, *p* = 0.008) groups were separate with close distance, the LI/HI (0.25 < R < 0.75, *p* < 0.05) were strongly overlapping. R is the statistic of ANOSIM test. There was the greater the difference between groups when the R value is closer to 1. This revealed that the LI/HI had a high similarity and the WR/ST had a low similarity. Interestingly, while the three omnivorous fish samples and environmental samples were analyzed separately, the results showed that the interindividual (HI, LI and MI) were strongly clustered together (Figure 2B), but the water and sediments groups were still separated from each other (Figure 2C). These results indicated that there were significant differences in the microbiota between the fish and environment groups, and that the microbial communities in fish samples were significantly separated but strongly overlapping, which would be affected by the habitats.

**Figure 3:**
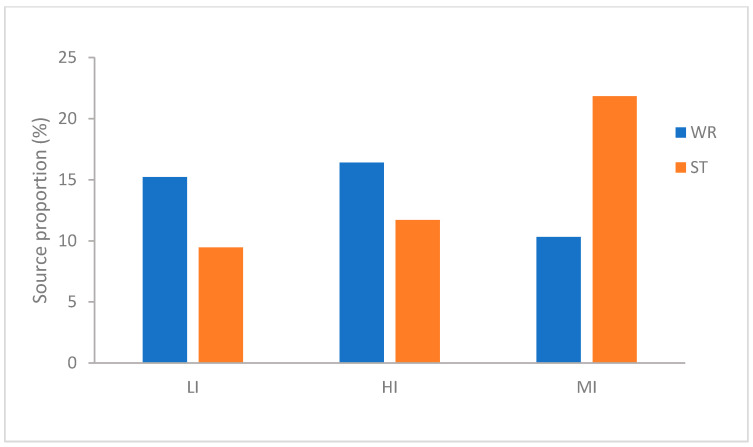
Percentage of intestinal microbial community of *T. houdemeri*, *H. leucisculus* and *O. mossambicus* derived from water and sediments by FEAST. HI, *T. houdemeri* intestines; LI, *H. leucisculus* intestines; MI, *O. mossambicus* intestines; ST, sediment; and WR, water.

To evaluate the contribution of habitat-associated microbiota to omnivorous fish intestine microbial communities, source tracking analysis was implemented. The three species of fish show different characteristics (Figure 3). As far as the MI, the microbiota showed greater derivation from the sediments (21.85%) than the water (10.32%). Interestingly, LI was similar to HI and exactly opposite to MI, the microbiota in LI and HI groups showed greater derivation from the water (*H. leucisculus* 15.23% and *T. houdemeri* 16.41%) than the sediments (*H. leucisculus* 9.46% and *T. houdemeri* 11.71%). These results indicated that the contribution of habitat characteristics to omnivorous fish intestinal microorganisms is different, the sediments has the greater contribution to MI than water. However the water has the more contribution to LI and HI than sediments.

**Figure 4:**
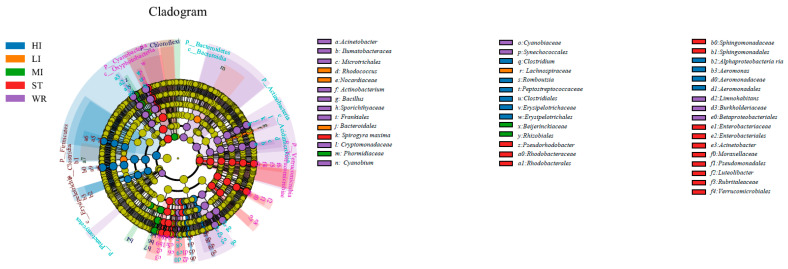
Linear effect size (LEfSe) analysis identified the most differentially abundant taxa (*p* < 0.05, LDA values > 4) in intestines, water, and sediment. Differentially abundant taxa of each group were distinguished by different colors (blue, orange, green, red and purple represented for HI, LI, MI, ST and WR., and yellow for non-significant). Inside-out radiating circles represent taxonomic levels from phylum to genus. HI, *T. houdemeri* intestines; LI, *H. leucisculus* intestines; MI, *O. mossambicus* intestines; ST, sediment; and WR, water.

LEfSe analysis was used to determine indicator taxa associated with the five groups (Figure 4). Across phylum to genus level, there were 10, 4, and 3 indicators of bacteria enriched in HI, LI and MI respectively, some lineages belonging to Firmicutes (at phylum level), Clostridiales (at order level), Nocardiaceae (at family level), Peptostreptococcaceae (at family level), *Cetobacterium* (at genus level) and *Clostridia* (at genus level). Moreover, across phylum to genus level, there were 14 indicators of bacteria enriched in ST, some lineages belonging to Cyanobacteria (at phylum level), Oxyphotobacteria (at phylum level), Enterobacteriales (at order level), Rhodobacteraceae (at family level). The water samples had the most numbers of indicators. Across phylum to genus level, there were 15 indicators of bacteria enriched in WR, some lineages belonging to Actinobacteria (at phylum level), Acidimicrobiia (at order level), Sporichthyaceae (at family level), *Cyanobium* (at genus level) among others.

### 3.2. Taxonomic Composition of Microbial Communities

**Figure 5:**
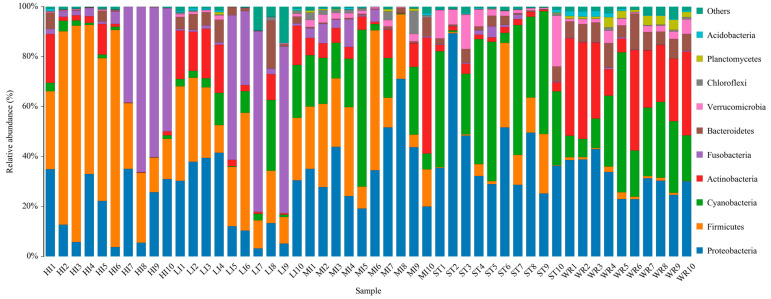
The composition and relative abundances of the bacterial community at phylum levels in intestines, water and sediment. Only the top 10 taxa with the largest average relative abundance are listed. HI, *T. houdemeri* intestines; LI, *H. leucisculus* intestines; MI, *O. mossambicus* intestines; ST, sediment; and WR, water.

Total bacterial in samples were classified into 42 phyla and 486 genera. The dominant bacterial phyla (top 10) of intestines, water, and sediments were Proteobacteria, Firmicutes, Cyanobacteria, Actinobacteria, Fusobacteria, Bacteroidetes, Verrucomicrobia, Chloroflexi, Planctomycetes, and Acidobacteria (Figure 5). Although the taxonomic composition of the microbial communities was similar among the five groups, the relative abundance of bacterial phyla was different. Proteobacteria was the most abundant phylum in MI, ST, and WR, and the second most abundant phylum in HI and LI. Firmicutes was the most abundance abundant phylum in HI and LI, accounting for 48.29% and 35.17%, respectively. Cyanobacteria was the second most abundant phylum in ST and WR, accounting for 37.29% and 29.43%, respectively. The abundances of Actinobacteria was higher in WR than other groups. The abundances of Chloroflexi was higher in ST than other groups. Fusobacteria showed high abundance in some samples of group HI and LI. The abundance of Planctomycetes in habitat samples (ST and WR) were clearly higher than intestinal samples (HI, LI and MI).

**Figure 6:**
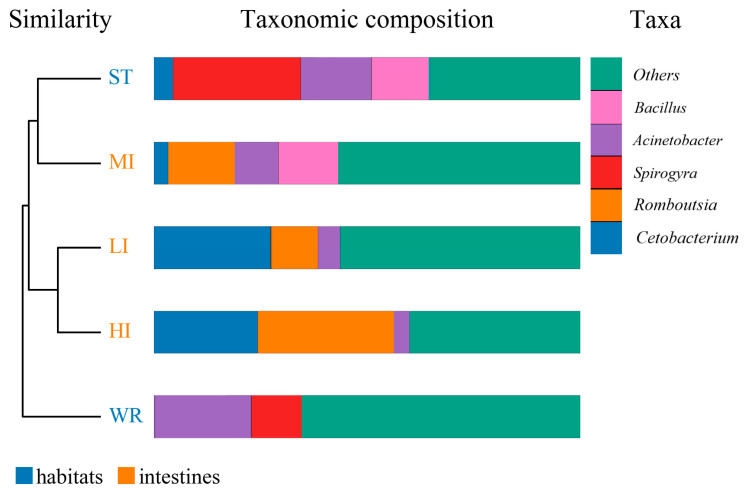
Cluster and relative abundances analysis of the bacterial community at genera levels in intestines, water, and sediment. Clustering based on the Bray-Curtis dissimilarity by UPGMA algorithm. Only the top five taxa with the largest average relative abundance are listed. HI, *T. houdemeri* intestines; LI, *H. leucisculus* intestines; MI, *O. mossambicus* intestines; ST, sediment; and WR, water.

**Figure 7:**
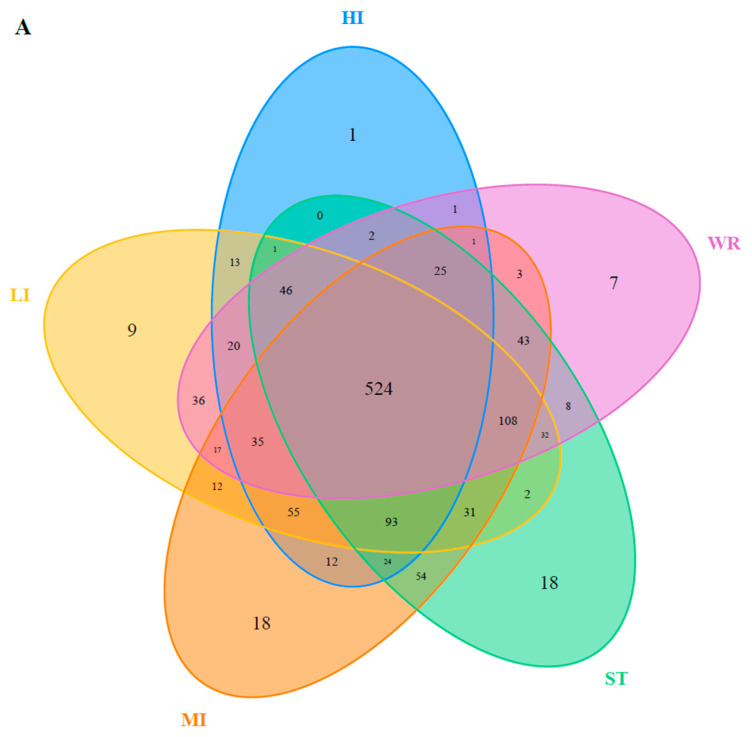

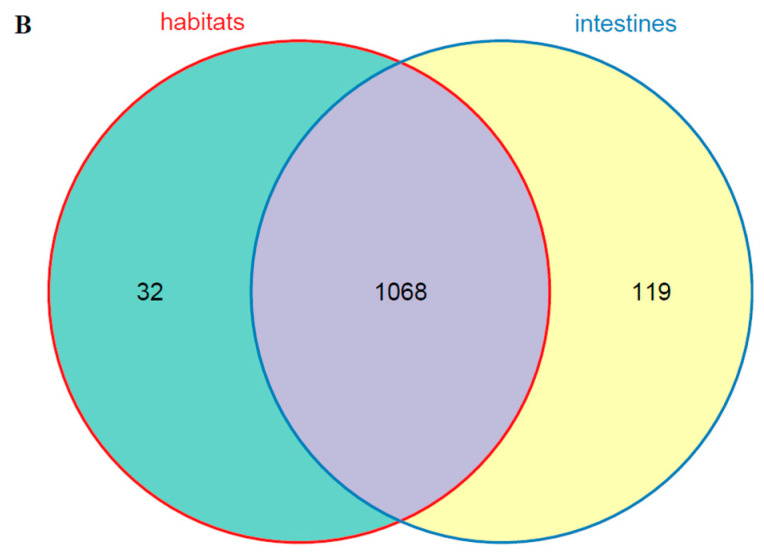
(**A**) Venn plot showing OTU overlap of HI, LI, MI, ST, and WR; (**B**) Venn plot showing overlap of habitats and intestines in microorganisms. HI, *T. houdemeri* intestines; LI, *H. leucisculus* intestines; MI, *O. mossambicus* intestines; ST, sediment; and WR, water.

The dominant bacterial genus (top five) of intestines, water, and sediments were displayed in Figure 6. The abundances of *Cetobacterium* were higher in the fish intestinal groups than in the habitat samples. The *Cetobacterium* was the dominant bacterial genus in HI, LI, and MI. The *Acinetobacter* and *Spirogyra* were the dominant bacterial genus in WR and ST, respectively. Furthermore, cluster analysis results showed that the bacterial communities of the HI and LI were clustered together, and however the bacterial communities of the MI and ST were clustered together (Figure 6), indicating that habitat characteristics and fish interspecific characteristics will affect fish intestinal bacterial communities. WR could be independent from the other groups based on the bacterial taxonomic composition. From the Venn diagram (Figure 7A), the OTUs numbers shared by the five groups were 524. The MI and ST groups had the largest number of unique OTUs, reaching 18 numbers, respectively. HI group had the lowest number of unique OTUs with only one. Moreover, from habitat and intestinal samples (Figure 7B), the intestine group (119) had more particular OTUs numbers than habitat group (32). These results indicate that: (1) the bacterial communities of the intestines, water and sediments were clearly different; and (2) not all microbes in water and sediments can be ingested and proliferate in omnivorous fish intestines.

### 3.3. Functional Prediction Differences of Microbial Community between Intestines and Habitats

**Figure 8:**
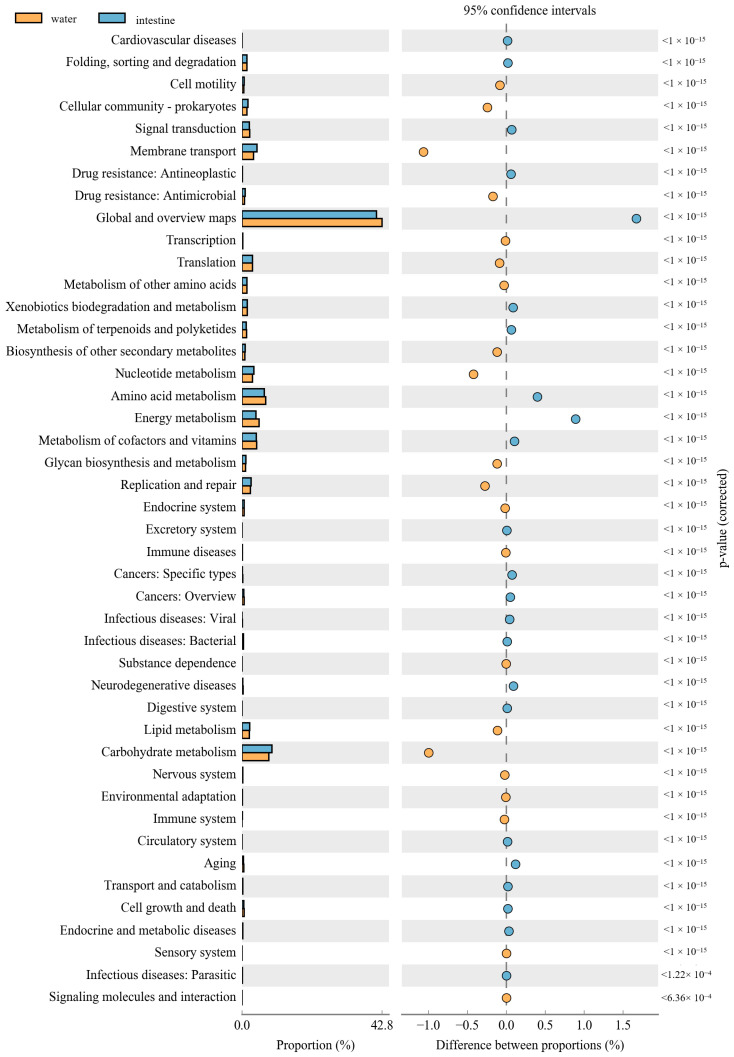
Function differences of the microbial community between water and intestines. Significantly different results (*p* < 0.05) among KEGG pathway categories are shown.

**Figure 9:**
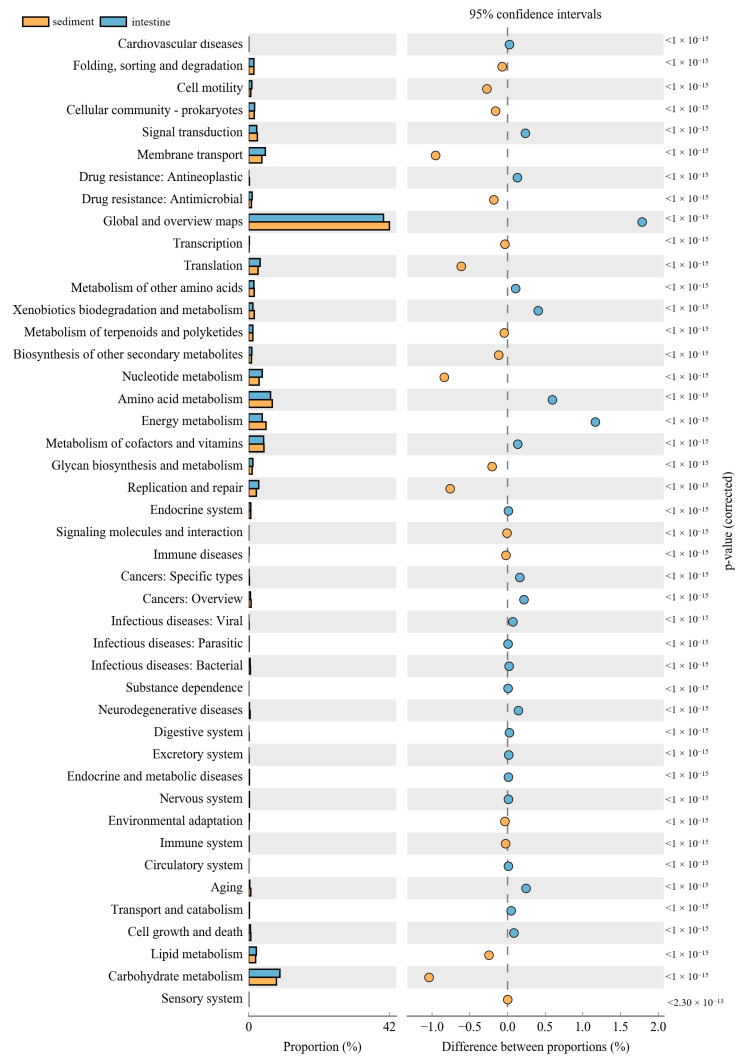
Function differences of the microbial community between sediments and intestines. Significantly different results (*p* < 0.05) among KEGG pathway categories are shown.

The PICRUSt was used to predict the function of 16S rRNA gene amplicons to analyze the function of the microbial community. Moreover, the NMDS results also indicated that the habitat microbiota (WR and ST) had significantly different functions than the fish intestines. The comparison of the microbial functions between intestines and water demonstrated 46 significantly changed categories (Figure 8). These results showed that “membrane transport”, “Nucleotide metabolism”, “Replication and repair”, “environmental adaptation” and “Carbohydrate metabolism” were significantly enriched in the intestines, compared with the WR group. However, most metabolism categories were significantly higher in the WR groups, including “Amino acid metabolism”, “Energy metabolism”, “Metabolism of cofactors and vitamins” and “Global and overview maps”. Additionally, the comparison of the microbial functions between intestines and sediments demonstrated 44 significantly changed categories (Figure 9). The results indicated that “Cell motility”, “Membrane transport”, “Immune system”, “Translation” and “Glycan biosynthesis and metabolism” were significantly enriched in the intestines, but the “Signal transduction”, “Metabolism of other amino acids”, “Xenobiotics biodegradation and metabolism” and “Energy metabolism” were significantly higher in sediment. Interestingly, some pathways of functions associated with diseases were selectively enriched in the WR (“Infectious diseases: Bacterial”, “Endocrine and metabolic diseases”, “Cancers”) or ST (“Infectious diseases: Parasitic” and “Infectious diseases: “Viral”). This result may show the immune effect of intestinal microorganisms on fish diseases.

## 4. Discussion

It is broadly accepted that microbiota plays an essential role in host nutrition, immunity, and health [67,68,69]. Recently, some studies have investigated the bacterial community composition in water [70], sediments [23], and fish intestines [24]. Furthermore, artificial fishery habitats have been widely used [71], especially for attracting the aggregation of omnivorous fishes [10,72,73]. However, there are few studies on the relationship between bacterial communities in omnivorous fish intestines and artificial fishery habitats. Here, we carried out comparative analysis of bacterial community associated with fish intestines, surrounding water, and sediments among three omnivorous fishes in artificial fishery habitats. The microbial communities of the intestines, water, and sediments samples exhibited significant differences in terms of diversity, composition, and predictive function.

Since aquatic animals are continuously exposed to water and sediment, the composition of their mucosal microbiota is strongly affected by the environmental microbiota, although there are significant differences between them [23,35,74]. The result of alpha diversity showed that microbial diversity of evenness and richness were significantly different in the habitat samples (ST, WR) than in the intestine samples (LI, HI, MI) of three omnivorous fishes, and the intestine microbial diversity of MI was higher than both LI and HI (Table 1). This result was consistent with the previous reports that microbial diversity in the intestines of large yellow croaker, silver carp, bighead carp, Chinese mitten crab, crayfish, and black sea bream were lower than that in their habitats (water and sediments) [20,35,45,75]. This also demonstrated that the microbial community of the water or sediments has a higher diversity, which could become the ideal habitat for various microbial communities related to the aquatic organism [76,77]. The fish intestinal microbiota may be limited by a variety of biological factors (such as host immune system and interspecific differences) and abiotic factors (such as nutrition source, dissolved oxygen, and water temperature) [19,78,79,80]. Thus, the microbial community of fish intestines samples can be different from the habitats’ samples [20,43,81]. The NMDS results showed that the bacterial beta-diversity of HI and LI were more similar to the water microbiota, but the bacterial beta-diversity of MI were more similar to the sediments microbiota (Figure 2). Additionally, the source tracking analysis showed that the sediments have a greater contribution to MI than water. However the water has a greater contribution to LI and HI than the sediments (Figure 3). It had been reported that intestine bacterial community of crayfish or crab was more closely related to the bacterial community of sediments [45,75]. This phenomenon may be related to the preference of aquatic organisms for habitat selection. The *O. mossambicus* generally lives in the bottom of the water and nest in the sediment, which characteristic was similar to the crayfish or crab. However the *T. houdemeri* and *H. leucisculus* are pelagic fishes [12,13,14,15]. Hence, the contribution of habitat characteristics to omnivorous fish intestinal microorganisms was different; the *T. houdemeri* and *H. leucisculus* came into contact with water more frequently than with sediments. However, the *O. mossambicus* has more connections with sediments than with water.

The microbial characteristics of intestines, water, and sediment were closely related to microbial compositions of samples. The dominant bacterial phyla were Proteobacteria, Firmicutes, Cyanobacteria, Actinobacteria, Fusobacteria, Bacteroidetes, Verrucomicrobia, Chloroflexi, Planctomycetes, and Acidobacteria in the HI, LI, MI, WR, and ST groups (Figure 5). Whether in the sea or fresh water, the Proteobacteria and Firmicutes are typical dominant bacteria related to fish intestines [80,82,83]. Previous reports had indicated that Firmicutes can promote caloric extraction of ingested food substances, affect nutrient acquisition and energy regulation [84,85], and Proteobacteria are often abundant in both healthy and diseased fish gut, which could be easy to colonize the intestine [34,86]. Although the taxonomic composition of the microbial communities was similar among the five groups, the relative abundance of bacterial phyla was different. At the genus level, the abundances of *Cetobacterium* were higher in the fish intestinal groups than in the habitat samples. The *Cetobacterium* was the dominant bacterial genus in HI, LI, and MI (Figure 6). Similarly, the LEfSe analysis also confirmed that *Cetobacterium* had been enriched in HI, LI, and MI, respectively, as one of the indicators. It had been reported that the *Cetobacterium* could serve as a potential candidate for probiotics [87,88]. Furthermore, the composition cluster analysis results showed that the bacterial communities of the HI and LI were clustered together, and however the bacterial communities of the MI and ST were clustered together (Figure 6), these results suggested that the intestines of omnivorous fish could selectively enrich specific taxa (which are potential probiotics) and also showed that habitats and fish interspecific characteristics will affect fish intestinal bacterial communities. The results also (Figure 7) showed that not all microbes in water and sediments can be ingested and proliferate in omnivorous fish intestines. Indeed, it was reported that the fish intestines was less aerobic than environmental water and sediments, and had immunological factors that may select specific types of bacteria [89,90,91,92].

The composition and balance of bacterial community will strongly affect the function of fish physiological processes [31,93]. These functional prediction results showed that “Environmental adaptation”, “Carbohydrate metabolism”, “Glycan biosynthesis and metabolism” and “Immune system” were significantly enriched in the intestines (Figure 8 and Figure 9). As mentioned above, this was consistent with the increase in the abundance of Proteobacteria, Firmicutes, and *Cetobacterium* in the fish intestine groups, which can affect fish health through the metabolism and immune system [32,87]. Interestingly, some pathways of functions associated with diseases were selectively enriched only in the WR (“Infectious diseases: Bacterial”, “Endocrine and metabolic diseases”, “Cancers”) or ST (“Infectious diseases: Parasitic” and “Infectious diseases: “Viral”), but without in intestines group. This result may also show the immune effect of intestinal microorganisms on fish diseases. Some pathways of functions still enriched in intestines group, such as “Membrane transport”, “Cell motility”, “Nucleotide metabolism”, “Replication and repair” and “Translation”. These predictive functions are usually related to the fact that the intestines are the main digestive and metabolic organ [82,94,95]. Absolutely, it is worth noting that the functional pathways were predicted by 16S data, and further functional verification should be studied in future.

## 5. Conclusions

This study is the first to analyze microbiota related to the intestines of omnivorous fishes, water, and sediments in artificial fishery habitats. In conclusion, we generated profiles of microbial communities in the water, sediments, and intestines of *T. houdemeri*, *H. leucisculus,* and *O. mossambicus*. It was evident that there were significant differences in the microbial communities among them. The microbial diversity in habitat samples (ST, WR) were significantly higher than in intestines samples (LI, HI, MI). The contribution of habitat characteristics to omnivorous fish intestinal microorganisms are different; the sediments has a greater contribution to *O. mossambicus* than water, but the water has a greater contribution to *T. houdemeri* and *H. leucisculus* than sediments. The bacterial community of intestines and habitats showed unique core indicators and predictive functions. Overall, these findings could enhance our understanding of the bacterial composition, diversity, and function of omnivorous fish intestines and habitats, and also provide fundamental information for the ecological benefits of artificial fishery habitats from the perspective of bacterial ecology.

## Figures and Tables

**Table 1:** Overview of the high-throughput read analysis, including OTUs and alpha-diversity statistics. The alpha-diversity analysis of bacterial community from intestines, water and sediments samples are shown at the level of OTUs. HI, *T. houdemeri* intestines; LI, *H. leucisculus* intestines; MI, *O. mossambicus* intestines; ST, sediment; and WR, water. OTUs are operation taxonomic units. The means ± SD data of Table 1 in the same row with different letters differ significantly (*p* < 0.05).

	HI	LI	MI	WR	ST
Total OTUs (97%)	853 ± 18 a	1034 ± 21 b	1055 ± 36 b	1011 ± 16 b	908 ± 15 a
Diversity indexes					
Shannon	3.1346 ± 0.4956 a	5.0506 ± 0.6294 b	5.4043 ± 0.3372 b	6.1935 ± 0.1496 c	4.5358 ± 0.3711 d
Gini-Simpson	0.6925 ± 0.0459 a	0.8312 ± 0.0627 b	0.9101 ± 0.0257 b	0.9625 ± 0.0036 c	0.8031 ± 0.036 d
Chao-1	1187 ± 51 a	1213 ± 50 a	1268 ± 43 a	1418 ± 34 b	1303 ± 36 c
ACE	1215 ± 37 a	1237 ± 39 a	1287 ± 26 a	1429 ± 29 b	1328 ± 21 c
Coverage	0.99 ± 0.01	0.99 ± 0.01	0.99 ± 0.01	0.99 ± 0.01	0.99 ± 0.01

**Table 2:** Analysis of similarity (ANOSIM) test of bacterial communities based on Bray–Curtis distances among intestines, water and sediment. HI, *T. houdemeri* intestines; LI, *H. leucisculus* intestines; MI, *O. mossambicus* intestines; ST, sediment; and WR, water. R is the statistic of ANOSIM test. There was the greater the difference between groups when the R value is closer to 1. The statistical difference between all pairs was significant (*p* < 0.05).

Group	R	*p* Value
LI/HI	0.2649	0.013
LI/MI	0.4218	0.008
HI/MI	0.4045	0.008
LI/WR	0.8681	0.005
LI/ST	0.9418	0.004
HI/WR	0.8734	0.002
HI/ST	0.9562	0.002
MI/WR	0.9847	0.001
MI/ST	0.8846	0.001
WR/ST	0.9925	0.001

## Data Availability

The data presented in this study are available on request from the corresponding author.
